# Split right coronary artery with split posterior descending artery

**DOI:** 10.34172/jcvtr.2023.31744

**Published:** 2023-03-16

**Authors:** Niraj Nirmal Pandey, Aprateem Mukherjee, Vineeta Ojha, Raghav Bansal, Sanjeev Kumar

**Affiliations:** ^1^Department of Cardiovascular Radiology & Endovascular Interventions, All India Institute of Medical Sciences, New Delhi-110029, India; ^2^Department of Cardiology, All India Institute of Medical Sciences, New Delhi-110029, India

**Keywords:** Coronary Artery Disease, Computed Tomography Angiography, Cardiac-Gated Imaging Techniques, Right Coronary Artery

## Abstract

We report a case of an 87-year-old man where coronary CT angiography incidentally demonstrated a "split" right coronary artery (RCA) featuring a "split" posterior descending artery. This case focusses on the morphological description of this variant as well as its differentiation from a "dual" or "duplicated" RCA.

## Case History

 An 87-year-old man, presenting with bilateral lower limb claudication and atypical chest pain, was referred for CT angiography for evaluation of the aorta and bilateral lower limb arteries along with evaluation of the coronary arteries. CT angiography revealed complete occlusion of the infra-renal abdominal aorta, bilateral common iliac arteries and bilateral external iliac arteries with distal reformation of the bilateral common femoral arteries.

**Figure 1:**
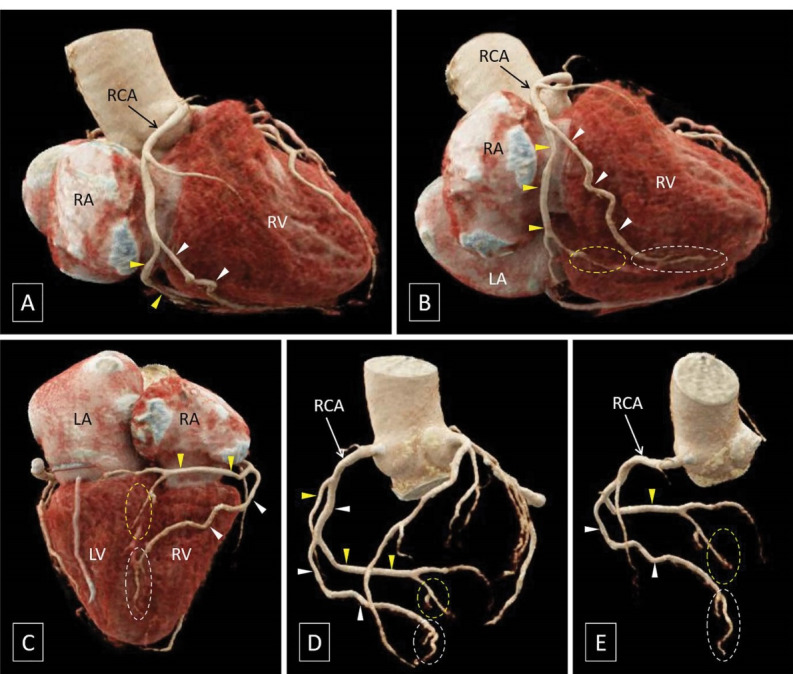
Volume rendered images monstrate the bifurcation of the mid-segment of right coronary artery (RCA) into a posterior branch (yellow arrowheads), which continued running in the right atrioventricular groove, and a good-sized anterior branch (white arrowheads), seen running over the right ventricular (RV) wall. The posterior branch reaches the crux of heart to give off the proximal part of the posterior descending artery (yellow dotted circle) while the anterior branch continues as the distal part of the posterior descending artery (white dotted circle). [RA: right atrium; LA: left atrium; LV: left ventricle]

 No obstructive atherosclerotic coronary artery disease was noted; incidentally, the mid-segment of the right coronary artery (RCA) split into a posterior branch, which continued running in the right atrioventricular (AV) groove, and a good-sized anterior branch, seen running over the right ventricular wall. The posterior branch of the RCA reached the crux of heart and gave off the posterior descending artery seen coursing in the proximal part of posterior interventricular groove. The anterior branch, however, also reached the posterior interventricular groove to continue as another posterior descending artery coursing in the distal part of posterior interventricular groove (Figure 1).

## Discussion

 A split RCA has been defined as an RCA featuring a split posterior descending artery with the length of each of the two posterior descending branches being variable.^1^ The anterior subdivision of the split RCA continues as the distal portion of the posterior descending artery (which supplies the distal aspect of the posterior septum and the inferior wall of the left ventricle) and may also give off the acute marginal artery which supplies the anterior free wall of the right ventricle. The posterior subdivision of the RCA remains in the AV groove and continues as the proximal portion of the posterior descending artery (which supplies the proximal aspect of the posterior septum).

 A ‘split’ RCA has previously been confused with a ‘dual’, ‘duplicated’ or ‘double’ RCA; however, a dual RCA features a single short proximal RCA trunk which divides into two parallel subdivisions of almost similar sizes which course closely together in the right AV groove, for the rest of their entire course and reach the crux of heart where both the anterior branch and posterior branch are seen to lead to a posterior descending artery respectively.^2,3^ On the contrary, in a split RCA, the anterior subdivision travels outside the right AV groove and does not reach the crux of the heart with the individual posterior descending branches supplying independent segments of the septum.

## Conclusion

 We reported a case of an 87-year-old man where coronary CT angiography incidentally demonstrated a “split” right coronary artery (RCA) featuring a “split” posterior descending artery. The case highlights the role of CT angiography in identifying such variant anatomy and in its differentiation from a “dual” or “duplicated” RCA.

## Competing Interests

 The authors declare that they have no conflict of interest.

## Ethical Approval

 Informed consent was obtained from the patient.

## Funding

 None.
